# Association between *NMD3* and symptoms of Parkinson’s disease in Chinese patients

**DOI:** 10.1186/s12883-019-1574-1

**Published:** 2020-01-14

**Authors:** Hui Wu, Hui Li, Zhiqiang Shi, Jiajia Tang, Shuya Mei, Tianyi Ai, Zhenzhou He

**Affiliations:** Department of Anesthesiology, South Campus, Ren Ji Hospital, School of Medicine, Shanghai Jiaotong University, 2000 Jiangyue Road, Shanghai, 201112 China

**Keywords:** Parkinson’s disease, *NMD3*, Single nucleotide polymorphism, Cognitive impairment

## Abstract

**Background:**

Parkinson’s disease (PD) is a progressive neurodegenerative movement disorder that is characterized by motor symptoms such as tremor, rigidity, slowness of movement and problems with gait. Large-scale meta-analyses of genome-wide association studies (GWAS) have identified few susceptibility loci in patients with sporadic PD. The aim of this study was to investigate the association between *NMD3* single nucleotide polymorphism (SNP) and symptoms in PD patients in South China.

**Methods:**

A total of 217 PD patients were recruited in this study and genotyped by using the SNaPshot technique and the polymerase chain reaction. All subjects were evaluated by the Mini-Mental State Examination (MMSE), Beijing version Montreal Cognitive Assessment (MoCA), Sniffin’ Sticks 16 (SS-16), Hamilton Anxiety Rating Scale, Hamilton Depression Rating Scale, 39-item Parkinson’s Disease Questionnaire (PDQ-39) and MDS Unified PD Rating Scale (MDS-UPDRS).

**Results:**

*NMD3* rs34016896 (C > T) carriers have worse cognitive function than wild types (MMSE: *p =* 0.042, *NMD3* wild type: 27.44 ± 2.89, *NMD3* carriers: 26.31 ± 3.79; MoCA: *p =* 0.005, *NMD3* wild type: 23.15 ± 4.20, *NMD3* carriers: 20.75 ± 6.68).

**Conclusions:**

The recessive and overdominant model of *NMD3* rs34016896 was associated with cognitive impairment in PD patients.

## Background

Parkinson’s disease (PD) is one of the most common neurodegenerative diseases, affecting approximately 1.7% of people over the age of 65, and the annual incidence ranges from 1.5 to 8.7/100,000 in the People’s Republic of China [1]. The pathological features of PD are the abnormal aggregation of α-synuclein and the loss of dopaminergic neurons in the substantia nigra [2]. Both acquired and inherited risk factors have been implicated in the death of dopaminergic neurons [3]. Genetic factors play a crucial role in the pathogenesis of sporadic PD. Genome-wide association studies (GWAS) have identified several susceptibility loci for PD [4–6]. Genes such as *LRRK2, SNC A,* etc. have been associated with the pathogenesis of PD [7, 8]. Marie Y. Davis reported that GBA variants predicted a more rapid progression of cognitive dysfunction and motor symptoms in patients with PD [9].

Recently, *variants at NMD3* were found to be related to substantia nigra neuronal loss and PD susceptibility [10, 11]. *The minor allele frequency of NMD3 rs34016896 was 0.41 in the Chinese PD population and 0.45 in the Chinese healthy population* [11]. *NMD3* encodes a ribosome-binding protein. Nmd3 is a structural mimic of eIF5A and activates the cpGTPase Lsg1 during 60S ribosome biogenesis [12]. Associations of *NMD3* rs34016896 with clinical pathological phenotypes have been discovered. JM Shulman and colleagues found that *NMD3* rs34016896 was related to the severity of nigral neuronal loss and not to Lewy bodies [13]. However, the function of *NMD3* in PD is unknown. In addition, it is necessary to investigate the association between *NMD3* and clinical symptoms of PD, which could indicate the pathogenesis of PD.

In this study, we attempted to discover the clinical hallmarks of *NMD3* rs34016896 (C > T) in southern Chinese PD patients.

## Methods

### Study population

PD patients were recruited from the outpatients clinic of Ren Ji Hospital (South Campus) and diagnosed by movement disorder specialists based on diagnostic criteria outlined by the Movement Disorders Society (MDS) [14]. For the PD patients, Hoehn-Yahr staging and their disease duration were recorded. A family history of PD was also recorded. Parkinsonism patients with secondary causes, such as inflammatory, drug-induced, vascular and toxin-induced parkinsonism, were excluded. Patients with parkinsonism with other neurodegenerative diseases, such as Wilson’s disease, progressive supranuclear palsy, cerebral-basal degeneration and multiple system atrophy, were also excluded. This study was approved by the ethics committee of Ren Ji Hospital.

### Evaluation

Each PD patient included in this study received an evaluation including the following rating scales: Unified PD Rating Scale provided by the Movement Disorders Society (MDS-UPDRS) was used to assess the status of PD [15]. The Mini-Mental State Examination (MMSE) and Beijing version of the Montreal Cognitive Assessment (MoCA) were adopted to assess cognitive function. The Sniffin’ Sticks 16 (SS-16) was used to assess olfactory function [16]. The Hamilton Anxiety Rating Scale and Hamilton Depression Rating Scale were used to assess anxiety and depression, respectively. The non-motor symptoms scale (NMSS) was used to assess non-motor symptoms. The Scales for Outcomes in Parkinson’s disease-Autonomic questionnaire (SCOPA-AUT) was used to assess autonomic symptoms. The 39-item Parkinson’s Disease Questionnaire (PDQ-39) was used to assess the quality of life of the PD patients. The researchers received strict training regarding the use of these scales before assessing the PD patients. We also documented the presence (yes/no) of the following symptoms that were assessed by two individual neurologists: hallucination, apathy, excessive daytime sleepiness, pain, frequent urination, constipation, postural hypotension, sialorrhea, restless legs syndrome (RLS), delusion, double vision, decreased attention, decreased recent memory, nycturia, sexual dysfunction, hypogeusia, change in weight, daytime sweatiness, nocturnal sweatiness, urgent urination or urinary incontinence, sensitivity to light, sensitivity to cold, sensitivity to hot, anxiety, depression, and probable rapid eye movement sleep behaviour disorder (RBD). Probable RBD was diagnosed via the RBD screening questionnaire [17]. We followed the method for detecting *NMD3* rs34016896 described by Li and colleagues [18].

### Statistics

The R stats (version 3.5.1) and CATT (version 2.0) packages were used to perform the statistical analyses. Student’s t tests were performed to evaluate the differences in numeric variables between *NMD3* carriers and wild types. For comparing categorical variables, chi-square tests were performed. The Cochran-Armitage test was used to assess ordinal variables between *NMD3* carriers and wild types and an additive model of *NMD3*. Logistic regression was used to assess the association between the additive model/dominant model/recessive model/overdominant model of *NMD3* and clinical phenotypes. The odds ratio (OR) and its 95% confidence intervals (CI) were also used. We also adjusted for age, sex and Hoehn-Yahr staging results.

## Results

There were 217 PD patients included in this study. SNPs of two PD patients failed to be detected. In all, there were 39 *NMD3* wild types and 178 *NMD3* carriers in our study. The minor allele frequency in our group was 0.41, which is similar to a previous genetic association study in southeastern China [11]. There was no difference in age, sex, disease duration, family history, and Hoehn-Yahr staging between the two groups. In the *NMD3* wild-type group, the age was 55.69 ± 10.24 years (mean ± SD), and 17 (43.59%) females were included in this group. In the *NMD3* carrier group, the average age was 57.03 ± 10.16 years (mean ± SD), and 73 (41.01%) females were included in this group. There were no differences in the total scores on the NMSS, SCOPA-AUT, or PDQ-39 between the two groups. Cognitive function assessed by the MMSE and MoCA of the *NMD3* wild types was better than the *NMD3* carriers (MMSE: *p =* 0.042, *NMD3* wild type: 27.44 ± 2.89, *NMD3* carriers: 26.31 ± 3.79; MoCA: *p =* 0.005, *NMD3* wild type: 23.15 ± 4.20, *NMD3* carriers: 20.75 ± 6.68) (Table 1).

**Table 1:** Demographic data and symptoms in PD patients involved in this study

	*NMD3* carriers (*n* = 178)	*NMD3* wildtypes (*n* = 39)	*p* value
Age, mean (SD)	57.03 (10.16)	55.69 (10.24)	0.461
Gender, female, N (%)	73 (41.01)	17 (43.59)	0.907
Disease duration, mean (SD)	4.79 (4.28)	5.56 (3.89)	0.275
Family history, N (%)	17 (9.55)	5 (12.82)	0.550
Hoehn – Yahr staging, N (%)			0.869
1.0	41 (23.03)	10 (25.64)	
1.5	29 (16.29)	4 (10.26)	
2.0	60 (33.71)	14 (35.90)	
2.5	32 (17.98)	7 (17.95)	
3.0	12 (6.74)	4 (10.26)	
4.0	4 (2.24)	0 (0.00)	
5.0	0 (0.00)	0 (0.00)	
MDS-UPDRS, mean (SD)	48.34 (27.10)	43.05 (20.51)	0.175
Part I	8.66 (6.16)	7.51 (5.43)	0.249
Part II	11.60 (7.71)	10.08 (5.81)	0.168
Part III	28.08 (17.45)	25.46 (14.10)	0.318
NMSS, mean (SD)	36.23 (35.57)	31.03 (27.42)	0.315
SCOPA-AUT, mean (SD)	10.92 (9.06)	9.08 (8.56)	0.233
PDQ-39, mean (SD)	19.31 (17.84)	16.23 (13.93)	0.241
MMSE, mean (SD)	26.31 (3.79)	27.44 (2.89)	0.042
MoCA, mean (SD)	20.75 (6.68)	23.15 (4.20)	0.005

MDS, movement disorders society; MMSE, Mini-Mental State Examination; MoCA, Montreal Cognitive Assessment; NMSS, non-motor symptoms scale; SCOPA-AUT, scales for Outcomes in Parkinson’s disease – autonomic questionnaire; SD, standard deviation; PD, Parkinson’s disease; PDQ-39, 39-item Parkinson’s disease Questionnaire; UPDRS, unified Parkinson’s disease rating scale

The presence of hallucination, postural hypotension, and delusion were associated with the additive model (hallucination: *p =* 0.025; postural hypotension: *p =* 0.007; and delusion: *p =* 0.038). In addition, trends in the presence of apathy, decreased recent memory and weight change were found under the additive model (apathy: *p =* 0.092; decreased recent memory: *p =* 0.064; and change in weight: *p =* 0.073) (Table 2).

**Table 2:** The association between symptoms and genetic models (additive and dominant models) of *NMD3* rs34016896

	Additive model	Dominant model			Dominant model (adjusted)*		
		*p* value	OR	95% CI	*p* value	OR	95% CI
Hallucination	0.025	0.368	1.78	(0.58, 7.79)	0.341	1.86	(0.59, 8.26)
Apathy	0.092	0.941	0.97	(0.49, 1.97)	0.955	0.98	(0.48, 2.01)
Excessive daytime sleepiness	0.444	0.908	0.95	(0.38, 2.15)	0.896	0.94	(0.38, 2.15)
Pain	0.696	0.474	0.77	(0.37, 1.56)	0.513	0.78	(0.37, 1.60)
Frequent urination	0.639	0.491	1.28	(0.64, 2.58)	0.526	1.27	(0.60, 2.72)
Constipation	0.930	0.656	0.85	(0.42, 1.71)	0.528	0.78	(0.36, 1.66)
Postural hypotension	0.007	0.052	2.38	(1.05, 6.16)	0.050	2.43	(1.05, 6.38)
Sialorrhea	0.487	0.600	1.20	(0.60, 2.42)	0.617	1.22	(0.56, 2.62)
RLS	0.905	0.266	0.66	(0.32, 1.40)	0.259	0.65	(0.31, 1.40)
Delusion	0.038	0.557	1.58	(0.42, 10.33)	0.605	1.51	(0.38, 10.10)
Double vision	0.197	0.144	3.02	(0.85, 19.31)	0.133	3.22	(0.86, 21.14)
Decreased attention	0.124	0.570	1.27	(0.58, 3.00)	0.554	1.29	(0.57, 3.14)
Decreased recent memory	0.064	0.227	1.54	(0.76, 3.09)	0.232	1.54	(0.76, 3.13)
Nocturia	0.870	0.845	1.08	(0.47, 2.34)	0.932	1.04	(0.43, 2.36)
Sexual dysfunction	0.188	0.272	1.64	(0.71, 4.27)	0.254	1.69	(0.72, 4.48)
Hypogeusia	0.202	0.827	1.08	(0.54, 2.22)	0.859	1.07	(0.53, 2.19)
Change of weight	0.073	0.323	0.49	(0.13, 2.38)	0.298	0.47	(0.12, 2.29)
Daytime sweatiness	0.449	0.964	1.02	(0.49, 2.17)	0.918	1.04	(0.49, 2.29)
Nocturnal sweatiness	0.405	0.847	0.93	(0.46, 1.96)	0.874	0.94	(0.45, 2.04)
Urgent urination or urinary incontinence	0.724	0.891	1.05	(0.52, 2.10)	0.946	1.03	(0.48, 2.18)
Sensitive to light	0.142	0.387	2.50	(0.47, 46.44)	0.381	2.55	(0.46, 47.88)
Sensitive to cold	0.484	0.589	1.30	(0.53, 3.66)	0.588	1.30	(0.53, 3.68)
Sensitive to hot	0.481	0.311	0.63	(0.27, 1.62)	0.283	0.60	(0.25, 1.59)
Anxiety	0.155	0.404	1.49	(0.62, 4.19)	0.335	1.64	(0.63, 4.86)
Depression	0.935	0.644	1.35	(0.43, 5.98)	0.664	1.34	(0.41, 6.09)
RBD	0.957	0.542	0.80	(0.39, 1.67)	0.498	0.77	(0.37, 1.65)
Olfactory dysfunction	0.784	0.845	1.08	(0.47, 2.34)	0.934	1.03	(0.44, 2.26)

CI, confidence interval; OR, odds ratio; RBD, rapid eye movement sleep behavior disorder; RLS, restless legs syndrome

^*^adjusted by age, gender and Hoehn-Yahr staging

Under the dominant model, the presence of postural hypotension was found (*p =* 0.052, OR: 2.38, CI: 1.05–6.16, before adjustment; *p =* 0.050, OR: 2.43, CI: 1.05–6.38, after adjustment) (Table 2).

Under the recessive model, the presence of hallucination, apathy, postural hypotension and delusion were found (hallucination: *p =* 0.012, OR: 2.96, CI: 1.29–7.10, before adjustment; *p =* 0.014, OR: 2.95, CI: 1.26–7.21, after adjustment; apathy: *p =* 0.011, OR: 2.06, CI: 1.18–3.61, before adjustment; *p =* 0.012, OR: 2.07, CI: 2.28–3.67, after adjustment; postural hypotension: *p =* 0.017, OR: 2.04, CI: 1.14–3.68, before adjustment; *p =* 0.016, OR: 2.08, CI: 1.15–3.82, after adjustment; delusion: *p =* 0.014, OR: 3.94, CI: 1.38–12.92, before adjustment; *p =* 0.012, OR: 4.41, 1.46–15.47, after adjustment). Trends for decreased attention, decreased recent memory, hypogeusia and change in weight were associated with the recessive model (decreased attention: *p =* 0.068, OR: 1.76, CI: 0.58–3.02, before adjustment; *p =* 0.075, OR: 1.78, CI: 0.94–3.38, after adjustment; decreased recent memory: *p =* 0.075, OR: 1.68, CI: 0.96–3.00, before adjustment; *p =* 0.069, OR: 1.71, CI: 0.96–3.09, after adjustment; hypogeusia: *p =* 0.086, OR: 1.63, CI: 0.93–2.85, before adjustment; *p =* 0.080, OR: 1.65, CI: 0.94–2.89, after adjustment; change in weight: *p =* 0.097, OR: 0.17, CI: 0.01–0.94, before adjustment; *p =* 0.089, OR: 0.16, CI: 0.01–0.90, after adjustment) (Table 3).

**Table 3:** The association between symptoms and genetic models (recessive models) of *NMD3* rs34016896

	Recessive model			Recessive model (adjusted)*		
	*p* value	OR	95% CI	*p* value	OR	95% CI
Hallucination	0.012	2.96	(1.29, 7.10)	0.014	2.95	(1.26, 7.21)
Apathy	0.011	2.06	(1.18, 3.61)	0.012	2.07	(2.28, 3.67)
Excessive daytime sleepiness	0.221	1.55	(0.78, 3.19)	0.213	1.56	(0.79, 3.23)
Pain	0.252	1.39	(0.79, 2.46)	0.286	1.36	(0.77, 2.42)
Frequent urination	0.879	1.04	(0.60, 1.81)	0.916	1.03	(0.57, 1.86)
Constipation	0.629	1.15	(0.66, 2.00)	0.649	1.15	(0.64, 2.07)
Postural hypotension	0.017	2.04	(1.14, 3.68)	0.016	2.08	(1.15, 3.82)
Sialorrhea	0.536	1.19	(0.68, 2.09)	0.552	1.20	(0.65, 2.23)
RLS	0.480	1.24	(0.68, 2.26)	0.520	1.22	(0.66, 2.26)
Delusion	0.014	3.94	(1.38, 12.92)	0.012	4.41	(1.46, 15.47)
Double vision	0.480	1.34	(0.58, 3.02)	0.420	1.43	(0.59, 3.40)
Decreased attention	0.068	1.76	(0.96, 3.26)	0.075	1.78	(0.94, 3.38)
Decreased recent memory	0.075	1.68	(0.96, 3.00)	0.069	1.71	(0.96, 3.09)
Nocturia	0.930	1.03	(0.55, 1.97)	0.940	1.02	(0.52, 2.03)
Sexual dysfunction	0.281	1.41	(0.75, 2.63)	0.317	1.39	(0.73, 2.63)
Hypogeusia	0.086	1.63	(0.93, 2.85)	0.080	1.65	(0.94, 2.89)
Change of weight	0.097	0.17	(0.01, 0.94)	0.089	0.16	(0.01, 0.90)
Daytime sweatiness	0.247	0.71	(0.39, 1.27)	0.176	0.65	(0.35, 1.20)
Nocturnal sweatiness	0.280	0.72	(0.40, 1.29)	0.199	0.67	(0.36, 1.23)
Urgent urination or urinary incontinence	0.677	1.12	(0.65, 1.96)	0.718	1,12	(0.62, 2.03)
Sensitive to light	0.151	2.38	(0.73, 8.28)	0.163	2.37	(0.71, 8.50)
Sensitive to cold	0.541	1.24	(0.61, 2.49)	0.561	1.23	(0.60, 2.47)
Sensitive to hot	0.809	0.91	(0.41, 1.94)	0.768	0.88	(0.39, 1.96)
Anxiety	0.149	1.63	(0.83, 3.20)	0.143	1.72	(0.83, 3.58)
Depression	0.626	0.79	(0.29, 1.99)	0.581	0.76	(0.27, 1.99)
RBD	0.573	1.18	(0.66, 2.10)	0.591	1.18	(0.65, 2.14)
Olfactory dysfunction	0.574	0.83	(0.45, 1.58)	0.593	0.84	(0.44, 1.61)

CI, confidence interval; OR, odds ratio; RBD, rapid eye movement sleep behavior disorder; RLS, restless legs syndrome

^*^adjusted by age, gender and Hoehn-Yahr staging

Under the overdominant model, an association with apathy and delusion were found (apathy: *p =* 0.011, OR: 2.05, CI: 1.19–3.57, before adjustment; *p =* 0.012, OR: 2.06, CI: 1.18–3.64, after adjustment; delusion: *p =* 0.048, OR: 3.66, CI: 1.14–16.31, before adjustment; *p =* 0.035, OR: 4.35, CI: 1.25–20.99, after adjustment.). A trend for an association between hallucination and the overdominant model was found (*p =* 0.071, OR: 2.32, CI: 0.97–6.17, before adjustment; *p =* 0.086, OR: 2.26, CI: 0.93–6.13, after adjustment) (Table 4).

**Table 4:** The association between symptoms and genetic models (overdominant models) of *NMD3* rs34016896

	Overdominant model			Overdominant model (adjusted)*		
	*p* value	OR	95% CI	*p* value	OR	95% CI
Hallucination	0.071	2.32	(0.97, 6.17)	0.086	2.26	(0.93, 6.13)
Apathy	0.011	2.05	(1.19, 3.57)	0.012	2.06	(1.18, 3.64)
Excessive daytime sleepiness	0.198	1.54	(0.80, 2.97)	0.186	1.56	(0.81, 3.03)
Pain	0.094	1.60	(0.92, 2.76)	0.121	1.55	(0.89, 2.70)
Frequent urination	0.700	0.90	(0.53, 1.54)	0.698	0.89	(0.50, 1.59)
Constipation	0.413	1.25	(0.73, 2.15)	0.349	1.32	(0.74, 2.36)
Postural hypotension	0.414	1.27	(0.71, 2.30)	0.407	1.29	(0.71, 2.36)
Sialorrhea	0.841	1.06	(0.61, 1.82)	0.845	1.06	(0.58, 1.93)
RLS	0.121	1.62	(0.89, 3.01)	0.131	1.62	(0.87, 3.06)
Delusion	0.048	3.66	(1.14, 16.31)	0.035	4.35	(1.25, 20.99)
Double vision	0.625	0.82	(0.36, 1.85)	0.669	0.83	(0.35, 1.98)
Decreased attention	0.176	1.53	(0.83, 2.88)	0.195	1.53	(0.81, 2.97)
Decreased recent memory	0.416	1.25	(0.73, 2.17)	0.391	1.28	(0.73, 2.23)
Nocturia	0.949	0.98	(0.52, 1.83)	0.998	1.00	(0.51, 1.94)
Sexual dysfunction	0.839	1.07	(0.57, 2.00)	0.928	1.03	(0.54, 1.97)
Hypogeusia	0.131	1.53	(0.88, 2.66)	0.114	1.56	(0.90, 2.73)
Change of weight	0.304	0.51	(0.13, 1.83)	0.295	0.50	(0.12, 1.82)
Daytime sweatiness	0.243	0.71	(0.40, 1.26)	0.157	0.65	(0.36, 1.18)
Nocturnal sweatiness	0.363	0.77	(0.44, 1.35)	0.254	0.71	(0.39, 1.28)
Urgent urination or urinary incontinence	0.763	1.09	(0.63, 1.86)	0.762	1.09	(0.61, 1.97)
Sensitive to light	0.456	1.60	(0.49, 6.14)	0.481	1.58	(0.46, 6.28)
Sensitive to cold	0.857	1.07	(0.53, 2.16)	0.880	1.06	(0.53, 2.15)
Sensitive to hot	0.582	1.24	(0.59, 2.69)	0.579	1.25	(0.57, 2.84)
Anxiety	0.442	1.30	(0.67, 2.60)	0.489	1.30	(0.63, 2.75)
Depression	0.405	0.68	(0.27, 1.69)	0.376	0.65	(0.25, 1.69)
RBD	0.306	1.35	(0.76, 2.40)	0.292	1.38	(0.76, 2.51)
Olfactory dysfunction	0.483	0.80	(0.42, 1.49)	0.555	0.82	(0.43, 1.56)

CI, confidence interval; OR, odds ratio; RBD, rapid eye movement sleep behavior disorder; RLS, restless legs syndrome

^*^adjusted by age, gender and Hoehn-Yahr staging

We performed the Bonferroni correction following multiple comparisons to adjust *p* values. After the correction, there were no remaining statistically significant results.

## Discussion

Our study found that *NMD3* carriers had worse cognitive function. The additive model of *NMD3* was associated with hallucination, postural hypotension and delusion. The dominant model of *NMD3* was associated with postural hypotension. The recessive model of *NMD3* was associated with hallucination, apathy, postural hypotension and delusion. The overdominant model of *NMD3* was associated with apathy and delusion. Trends for associations between the additive model of *NMD3* and apathy, decreased recent memory and change in weight were found. Trends for associations between the recessive model of *NMD3* and decreased attention, decreased recent memory, hypogeusia and change in weight were found. Trends for associations between the overdominant model of *NMD3* and hallucination were found. To our knowledge, this is the first study to investigate the association between *NMD3* and its clinical symptoms in Chinese PD patients.

*NMD3* encodes a cytoplasmic protein for stable 60S ribosomal subunits [19, 20]. The function of ribosomes in the pathogenesis of PD remains unknown. A study revealed that parkin–PARIS (parkin-interacting substrate) played a deleterious role in rRNA transcription in PD patients, which indicated that ribosomes might be involved in the pathogenesis of PD [21]. A possible hypothesis of the function of *NMD3* is that the dysfunction or dysregulation of ribosomes produces proteins relevant to PD. More relationships between the eukaryotic ribosome and PD should be discovered.

JM Shulman and colleagues found that *NMD3* rs34016896 was associated with nigral neuronal loss and not with Lewy bodies [13]. This research indicated that *NMD3* rs34016896 was associated with nigral neurodegeneration rather than the formation of Lewy bodies. Neuronal loss, especially dopaminergic neuronal loss, is associated with the pathogenesis of PD. However, we have little information regarding the details of the type of neurons for neuronal loss. To date, there have been no studies on the clinical phenotype based on the detailed pathological phenotypes. It is difficult to elucidate the impact of neuronal loss on clinical phenotypes. Further research on NMD3-related neuronal loss could uncover the presence of those relevant symptoms.

The strengths of our study are that PD were assessed by a structured scale, which is widely accepted. The diagnosis was based on MDS criteria. We also covered a wide-range assessment examining motor function, non-motor symptoms and quality of life in PD patients.

This study has some weaknesses and limitations. First, we did not perform objective clinical methods, such as electrophysiology, to assess symptoms. Second, we did not perform stratifications due to the small sample size. Third, the sample of our study was small, and our study was a single-centre study. More multicentre and larger studies are warranted.

## Conclusions

In conclusion, *NMD3* carriers had worse cognitive function. The additive model of *NMD3* was associated with hallucination, postural hypotension and delusion. The dominant model of *NMD3* was associated with postural hypotension. The recessive model of *NMD3* was associated with hallucination, apathy, postural hypotension and delusion. The overdominant model of *NMD3* was associated with apathy and delusion. More larger and multicentre studies are warranted. The recessive and overdominant model of *NMD3* rs34016896 was associated with cognitive impairment in PD patients.

## Data Availability

The datasets used and/or analysed during the current study are available from the corresponding author on reasonable request.

## Abbreviations

CI: confidence interval
MDS: Movement Disorders Society
MMSE: Mini-Mental State Examination
MoCA: Montreal Cognitive Assessment
NMSS: non-motor symptoms scale
OR: odds ratio
PARIS: parkin-interacting substrate
PD: Parkinson’s disease
PDQ-39: 39-item Parkinson’s Disease Questionnaire
RBD: rapid eye movement sleep behaviour disorder
RLS: restless legs syndrome
SCOPA-AUT: Scales for Outcomes in Parkinson’s disease-Autonomic questionnaire
SS-16: Sniffin’ Sticks 16
UPDRS: Unified Parkinson’s Disease Rating Scale
